# Dynamic range optimization for treatment time reduction in respiratory‐gated proton therapy using RayStation v2025

**DOI:** 10.1002/acm2.70510

**Published:** 2026-02-16

**Authors:** Sungkoo Cho, Jung Il Yu, Hee Chul Park, Kwanghyun Jo

**Affiliations:** ^1^ Department of Radiation Oncology Samsung Medical Center Seoul Republic of Korea

**Keywords:** beam‐on time efficiency, dynamic range, intensity‐modulated proton therapy, liver cancer, respiratory gating

## Abstract

**Purpose:**

This study introduces dynamic range optimization, which constrains the lower limit of the minimum MU per spot, as a planning‐based strategy to enhance efficiency in respiratory‐gated proton therapy and evaluates the resultant dosimetric trade‐offs.

**Methods:**

We analyzed 101 hepatocellular carcinoma patients who received line‐scanning proton therapy. We developed a computational framework to predict the total treatment time, integrating dynamic range‐mediated changes in beam‐on time (BoT) with respiratory gating dynamics. The model was validated against fully dynamic range‐optimized plans generated by RayStation v2025 across five dynamic range values (no constraint, 200, 100, 50, and 10). These values represent the ratio of maximum to minimum MU allowed within an energy layer; specifically, a lower dynamic range value imposes a stricter constraint by elevating the minimum MU floor, thereby reducing intensity modulation flexibility while increasing delivery speed. Dosimetric trade‐offs were quantified using fully re‐optimized plans for a representative case under dynamic range = 100 and 10 constraints. The study used a layer switching time (T_LS_) of 2 s and amplitude‐based respiratory gating. A RayStation‐integrated Python script was developed for clinical decision support.

**Results:**

Decreasing the dynamic range systematically reduced beam‐on time in a sigmoid‐shape pattern. The computational model's predictions for beam‐on time showed a maximum error of 10.4% compared to RayStation v2025 calculations, confirming its accuracy. Total treatment time reduction reached a plateau at certain dynamic range thresholds, particularly when layer delivery plus switching was completed within a single gating‐on period, indicating no further efficiency gain from additional dynamic range reduction. While target coverage remained equivalent across dynamic range values, lower dynamic range values systematically increased normal tissue doses due to reduced intensity modulation capability, with organs proximal to the target experiencing the largest relative increases (e.g., gallbladder mean dose increased 16.4% at DR = 10).

**Conclusion:**

Dynamic range optimization is an effective, planning‐based method to reduce treatment time in respiratory‐gated proton therapy, operating independently of patient cooperation or system modifications. The developed RayStation‐integrated tool enables clinicians to identify the optimal patient‐specific dynamic range, balancing efficiency gains against acceptable dosimetric quality, particularly for organs‐at‐risk adjacent to targets. This approach provides a technical foundation for individualized dynamic range selection during treatment planning.

## 1 INTRODUCTION

Proton therapy for liver cancer has gained increasing clinical acceptance due to superior dose conformity compared to conventional photon therapy.^1^ However, respiratory motion management remains challenging, affecting both treatment accuracy and delivery efficiency. While various motion management strategies exist, respiratory gating has been clinically adopted because it provides an optimal balance between target coverage and normal tissue sparing. However, this approach substantially increases treatment time compared to free‐breathing delivery,^2^ creating a significant clinical challenge.

Prolonged treatment time in respiratory gating proton therapy arises from interruptions during inappropriate respiratory phases and energy layer switching delays that compound with respiratory interruptions. Energy layer switching time (T_LS_) varies widely across facilities, ranging from 0.2 to 6 s depending on the beam delivery system.^3^, ^4^, ^5^ While recent technological advancements have demonstrated that reducing T_LS_ can decrease total treatment time,^4^ such improvements require infrastructure investment.

The effort to reduce treatment time falls into two categories, each with fundamental limitations. Treatment‐system‐based approaches, including energy layer minimization,^6^, ^7^ increased spot spacing parameters,^8^ delivery pattern optimization,^9^ and modified beam sequencing algorithms,^5^, ^10^ require facility‐specific modifications and are difficult to deploy universally. Patient‐dependent approaches utilize respiratory training or audio‐visual guidance systems^11^ to reduce the amplitude of respiration or regularize the respiratory period. However, such methods are difficult to achieve in elderly patients, in those with respiratory comorbidities, or in patients experiencing treatment‐related anxiety.^12^ These limitations highlight an unmet clinical need: optimization strategies that operate entirely within the treatment planning process, independent of patient cooperation or system modifications.

RayStation v2025 has recently introduced dynamic range (DR) functionality for line scanning proton systems, offering a potential solution to this clinical gap. Dynamic range controls the range of intensity modulation by constraining minimum dose rate values within each energy layer. The user‐defined global dynamic range parameter of the beam constrains individual energy layers, adjusting the minimum MU threshold relative to the maximum MU specific to that layer. A lower dynamic range value narrows the window between the minimum and maximum monitor unit (MU), which in turn increases the minimum MU, thereby reducing beam‐on time while simultaneously reducing intensity modulation capability.^13^, ^14^ This planning‐based parameter enables direct control over the efficiency‐quality trade‐off during treatment plan optimization, without requiring patient cooperation or system modifications.

Our previous work^3^ demonstrated that treatment time efficiency in respiratory‐gated proton therapy depends on the synchronization between gating‐off periods and layer switching operations. Building on this foundation, the present study introduces dynamic range optimization as a planning‐based strategy to enhance efficiency through controlled modification of beam delivery patterns.

Dynamic range optimization adjusts minimum dose rate constraints during treatment planning, which alters beam‐on time per layer and consequently modifies the temporal synchronization between respiratory gating and layer switching. Notice that dynamic range operates only at the planning stage and does not alter the gating mechanism during beam delivery. In this study, respiratory gating remains active throughout treatment for motion management, while dynamic range determines how efficiently the dose is delivered during gating‐on periods.

This study addresses three objectives: First, we develop a computational framework integrating dynamic range effects with respiratory gating dynamics to predict treatment time across diverse breathing periods, enabling efficient dynamic range selection without exhaustive plan re‐optimization. Second, we quantify dosimetric trade‐offs between efficiency gains and plan quality using fully dynamic range‐optimized plans generated by RayStation v2025. Third, we provide a clinical decision‐support tool for patient‐specific dynamic range selection that systematically balances efficiency improvements against dosimetric criteria.

## 2 MATERIALS AND METHODS

### 2.1 Study design

This study evaluates dynamic range as a planning‐based optimization parameter for respiratory‐gated proton therapy through a four‐stage process separating computational prediction, experimental validation, and clinical applicability assessment.

xStage 1 develops a computational model that predicts dynamic range effects on treatment time under respiratory gating constraints. Extending our previous work,^3^ the model incorporates dynamic range‐mediated changes in beam‐on time and dose rate distribution, enabling efficient exploration of dynamic range parameter space across a large cohort (*N* = 101) with diverse respiratory patterns without exhaustive plan re‐optimization. Stage 2 validates computational predictions against fully dynamic range‐optimized plans generated in RayStation v2025. Since the computational model simulates dynamic range effects on existing plans while RayStation performs complete re‐optimization with active dynamic range constraints, agreement between predicted and optimized results confirms model accuracy. Stage 3 applies the validated model to characterize dynamic range effects across 101 patients with varying respiratory parameters, quantifying how efficiency gains depend on patient‐specific factors and establishing an empirical foundation for clinical dynamic range selection. Stage 4 evaluates dosimetric trade‐offs using a representative case fully re‐optimized under different dynamic range constraints to demonstrate how dynamic range constrains intensity modulation capability in dose distribution. Throughout all stages, amplitude‐based respiratory gating remains active for motion management.

This retrospective study analyzed 101 hepatocellular carcinoma patients who received proton therapy from January to December 2020 at our institution. All patients were treated using a Sumitomo Heavy Industry (SHI, Japan) cyclotron system with an energy range of 70–230 MeV and layer switching time (T_LS_) of 2 s. Treatment planning utilized 4D CT images acquired with a 590 RT scanner (GE Healthcare, Fairfield, CT), and amplitude‐based respiratory gating was applied during treatment delivery. Maximum intensity projection (MIP) images were generated for each treatment plan. Treatment plans employed two or three fields using single‐field uniform dose (SFUD) technique with 1.1 relative biological effectiveness (RBE). RayStation v6 (RaySearch AB, Stockholm, Sweden) served as the treatment planning system for the original treatment plans, while v2025 was employed for evaluating dynamic range implementation. Detailed characteristics of the treatment plans are provided in Table S1. The study was approved by the Institutional Review Board of Samsung Medical Center (2023‐07‐098‐001) with a waiver for written informed consent.

### 2.2 BoT calculational model with dynamic range effects

In line scanning proton therapy, each energy layer comprises multiple line segments with MU weights determined by intensity modulation requirements. The scanning system must maintain speeds accommodating the lowest‐weighted segment within each layer, and the speed cannot exceed the maximum scanning speed (Speed_max_). The minimum MU value within a layer thus constrains the dose rate for the entire layer.

Dynamic range addresses this constraint by establishing minimum MU thresholds within each energy layer.^13^ Elevating minimum MU values enables higher dose rates across all segments while reducing the capability to deliver low‐weighted segments. This creates a trade‐off between delivery speed and the range of intensity modulation. As a planning system constraint rather than a beam control parameter, dynamic range operates independently of patient respiratory behavior.

Our model simulates dynamic range effects on treatment plans through a four‐step process. First, we calculate the baseline dose rate for each energy layer as doserate_initial_ = (MU_min_ ÷ length_segment_) × Speed_max_, where MU_min_ represents the minimum MU value within the layer and Speed_max_ = 2000 cm/s. This step establishes the reference dose rate that would be achieved without dynamic range optimization. Second, we apply the dynamic range constraint by adjusting the dose rate based on the maximum MU: doserate_DR_ = (MU_max_ ÷ length_segment_) × Speed_max_ ÷ (dynamic range), where MU_max_ is the maximum MU value in the layer. The formulation reflects how dynamic range elevates the minimum MU threshold effectively, thereby increasing the dose rate by a factor related to the ratio of MU_max_ to MU_min_. Third, we validate that the calculated parameters remain within machine limits. If dynamic range‐adjusted dose rates would require speeds exceeding Speed_max_, we proportionally scale the MU values to satisfy this constraint. Fourth, we calculate the dynamic range‐adjusted beam‐on time for each line segment as BoT_DR_ = MU_adjusted_ ÷ doserate_DR_, where MU_adjusted_ represents the MU value after constraint validation. The total beam‐on time for each energy layer is the sum of BoT_DR_ across all line segments within the layer.

Our model is based on the observation that RayStation's dynamic range implementation primarily adjusts minimum MU values within existing line segments rather than fundamentally restructuring spot maps. This mechanism operates consistently regardless of patient‐specific anatomy. Validation against RayStation v2025 across five dynamic range values (Section 3.1) confirms this approach accurately reproduces RayStation's behavior within the dynamic range from 10 to 200.

Table 1 demonstrates this mechanism using a representative 189.6 MeV layer with five line‐segments (0.6 cm each), which is the first layer of patient 14. Without dynamic range, the layer dose rate is constrained to 3.3 MU/s by segment 5 (MU = 0.0010), yielding a total beam‐on time of 0.0138 s. The treatment plan with fully optimized by applying dynamic range = 10 in RayStation v2025 elevates the effective dose rate to 6.592 MU/s, reducing beam‐on time to 0.0072 s (52% reduction). Segments 1 and 5 require MU elevation to satisfy the dynamic range‐imposed minimum threshold, illustrating the intensity modulation trade‐off. RayStation v2025 performs full re‐optimization with dynamic range constraints throughout the planning process, potentially yielding different MU distributions while maintaining similar dose rate and beam‐on time relationships.

**TABLE 1:** Computational model prediction of dynamic range effects on beam‐on time for a representative energy layer (189.6 MeV, five segments, 0.6 cm each). dynamic range = 10 increases dose rate from 3.3 to 6.592 MU/s, reducing total beam‐on time by 52%. Segments 1 and 5 require MU elevation (bold) to satisfy dynamic range‐imposed minimum thresholds.

	No DR MU/s = 3.3		DR = 10 MU/s = 6.592	
	MU	BoT	MU_DR_	BoT_DR_
1	0.0013	0.0004	0.0020	0.0003
2	0.0198	0.0060	0.0198	0.0030
3	0.0178	0.0054	0.0178	0.0027
4	0.0056	0.0017	0.0056	0.0009
5	0.0010	0.0003	0.0020	0.0003

### 2.3 Computational framework for dynamic range‐mediated gated treatment time prediction

Establishing how dynamic range modifies beam delivery parameters at the individual layer level (Section 2.2), we now describe the computational framework that integrates these dynamic range effects with respiratory gating dynamics to predict total treatment time. This study extends our previous framework for respiratory‐gated proton therapy.^3^ While that work identified optimal breathing periods for maximizing gating efficiency under fixed beam delivery patterns, dynamic range introduces a controllable parameter in the planning stage, modulating beam‐on time.

Our investigation addresses two distinct questions requiring different methodological approaches. First, how does dynamic range affect treatment time for a given beam delivery pattern? This involves deterministic physical relationships between dynamic range constraints, dose rate, beam‐on time, and gating‐induced interruptions, which we model through computational simulation based on the beam delivery process. Second, how does dynamic range‐constrained optimization affect plan quality? This requires actual plan re‐optimization in RayStation v2025 (Section 2.5), as dose redistribution involves complex optimization algorithms, including spot weight adjustments and energy layer selection that cannot be reliably simulated by our model.

Our computational framework addresses the first question through physics‐based modeling with Python 3.10, validated against RayStation v2025 in Section 2.4. This approach enables efficient exploration of dynamic range parameter space across 101 patients and diverse respiratory periods because systematic evaluation through every case would require 800 plan re‐optimizations consuming over 400–600 h of computational time.

The framework integrates three modules modeling interactions between dynamic range, respiratory motion, and gating constraints. The first module simulates dynamic range effects on beam delivery by applying the algorithm from Section 2.2 to predict dynamic range‐mediated changes in dose rate and beam‐on time for each energy layer, processing existing spot maps and energy layer structures from RayStation v6‐optimized plans without full re‐optimization in v2025.

The second module generates realistic breathing patterns using our validated three‐component sinusoidal model:

(1) $$Sig\left(x\right)=\sin\left(x\right)+\sin^{2}\left(x+\frac{0.7}{\pi }\right)+\sin^{2}\left(x+\frac{\pi -5.4}{2}\right),$$

where *x* represents normalized time as *x* = 2 πt/*T_R_* and *T_R_* denotes the respiratory period.^3^ Amplitude‐based gating logic controls beam on/off timing based on whether the respiratory signal falls within predefined gating windows.

The third module integrates dynamic range‐adjusted beam‐on times with respiratory gating patterns to calculate total treatment time by sequentially processing each energy layer while tracking respiratory phase, determining layer switching timing during gating‐on periods, and accumulating dead time from gating‐off periods and layer switching delays.

Total treatment time comprises three components: beam‐on time (*T_BoT_*), energy layer switching time *T_LS_* × (*N_Layer_* – 1), and beam dead time (*T_dead_*) including all gating‐off periods (*T_off_*). The relationship follows:

(2) $$T_{total}=T_{BoT}+T_{LS}\times \left(N_{Layer}-1\right)+T_{dead},$$

where, *N_Layer_* represents the number of energy layers, and the subtraction reflects that no switching occurs after the final layer. This formulation has a limitation in capturing the complex interplay between layer switching timing and gating dynamics. Notably, when a layer switch coincides with a gating‐on phase, the interaction between *T_LS_* and *T_off_* contributes to a partial increase in the total treatment time. To address this, our simulation framework integrates these temporal dependencies into *T_dead_* by sequentially simulating each energy layer in synchronization with the respiratory phase.

The framework investigates how dynamic range‐mediated reductions in beam‐on time minimize total treatment time while accounting for timing interactions between shortened beam delivery and respiratory phase dynamics under unavoidable gating‐induced interruptions.

### 2.4 Model validation

Our computational model simulates dynamic range effects on treatment time based on beam delivery physics rather than replicating RayStation's optimization algorithm. Validation focuses on whether physics‐based predictions accurately capture dynamic range‐mediated changes in beam‐on time compared against fully optimized RayStation v2025 plans.

Our model development was guided by preliminary analysis of RayStation v2025 plans optimized with varying dynamic range constraints. This analysis revealed that dynamic range primarily operates by elevating minimum MU values within energy layers to increase dose rates. While RayStation's full optimization can redistribute proton fluences and modify line segment patterns, such fundamental restructuring occurs primarily when minimum MU elevation alone cannot satisfy dose constraints, which is a relatively infrequent scenario. Based on this observation, we implemented the simplified mechanism described in Section 2.2.

Validation focused on algorithmic accuracy rather than patient variability. Our model does not aim to replicate every aspect of RayStation's optimization algorithm but rather to accurately reproduce its dominant dynamic range mechanism. Dynamic range primarily increases treatment efficiency by elevating minimum MU values, which directly determine layer dose rates and beam‐on time. We hypothesized that redistribution of line‐segments due to the full optimization have minimal impact on beam‐on time. If validation through comparison of predicted versus actual beam‐on times across the full dynamic range confirms this hypothesis, it demonstrates our model captures the mechanistic essence of dynamic range effects governing treatment efficiency.

Comprehensive validation was employed for Patient 14, who has two fields. We generated five treatment plans in RayStation v2025 with identical dose constraints but different dynamic range values (no constraint, 200, 100, 50, and 10), comparing total beam‐on time between model predictions and RayStation v2025 calculations.

### 2.5 Clinical impact on dose distribution

With model accuracy established through algorithmic validation, we evaluated the dosimetric consequences of dynamic range‐constrained treatment plan using fully re‐optimization in RayStation v2025. We selected the same case of Table 1, Patient 14, for comprehensive dosimetric evaluation. We generated three independent treatment plans in RayStation v2025 with identical dose constraints and optimization objectives, differing only in dynamic range constraints: baseline (RayStation v6, no dynamic range constraint), dynamic range = 100 (fully re‐optimized in v2025), and dynamic range = 10 (fully re‐optimized in v2025). This design isolates dynamic range effects on plan quality from differences in optimization goals.

## 3 RESULTS

### 3.1 Model validation

Table 2 compares our model calculation and RayStation v2025 optimization on beam‐on time for two fields of the representative case. It demonstrates two critical validation points. First, our model accurately predicts beam‐on time across the entire dynamic range (maximum error 10.4%). Second, the consistency pattern confirms our model correctly implements RayStation's dynamic range logic.

**TABLE 2:** Validation of computational model against RayStation v2025. Plans originally optimized at specific dynamic range values were recalculated at different dynamic range settings. Identical values when applying a less restrictive dynamic range demonstrate accurate reproduction of RayStation's logic.

	Port 1			Port 2		
DR	RS v2025	Model	Diff.	RS v2025	Model	Diff.
200	43.7 s	43.7 s	0.0%	64.4 s	64.5 s	0.2%
100	36.4 s	37.8 s	3.8%	53.0 s	55.5 s	4.7%
50	18.3 s	20.2 s	10.4%	28.2 s	29.9 s	6.0%
10	10.7 s	10.7 s	0.0%	22.4 s	21.8 s	−2.7%
1		10.7 s			21.8 s	

In the extreme case of dynamic range = 1, there is no further beam‐on time reduction. This plateau reflects the physical limit where dose rate reaches the maximum value of the treatment machine, confirming our model correctly captures the machine constraints. For the dynamic range values from 10 to 200, treatment plans optimized in RayStation v2025 demonstrate systematic consistency: the number of layers remains unchanged, and the line segment configurations, including both their number (100–500) and positions, remain nearly identical across all plans, with only two layers differing by two segments (Figure S4). This systematic consistency across five dynamic range values confirms that our assumption in Section 2.2 holds, thereby validating that our computational approach faithfully reproduces RayStation's dynamic range algorithm. And the detailed analysis of beam‐on time reduction in treatment plans from RayStation v2025 was demonstrated in Figure S3.

### 3.2 Dynamic range effects on treatment time

**FIGURE 1:**
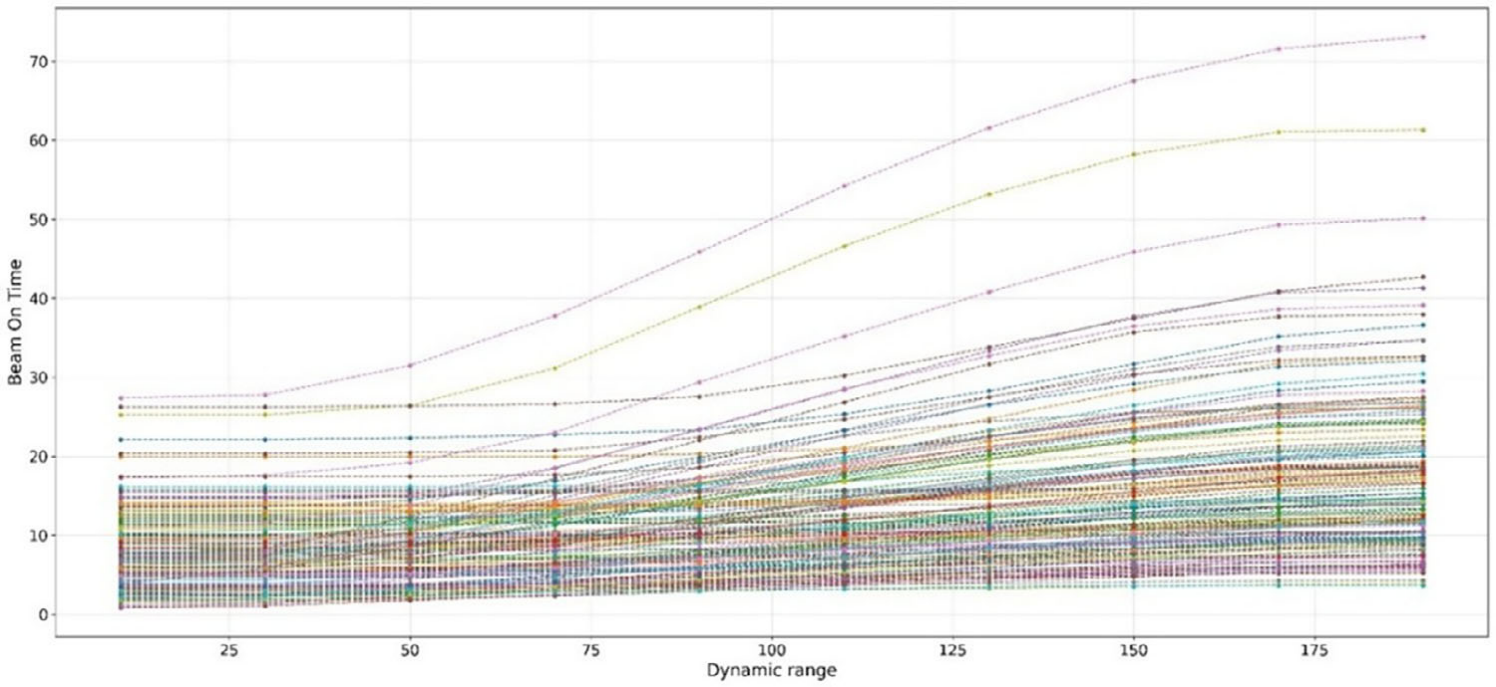
Beam‐on time as a function of dynamic range. Each line represents one treatment field calculated by our model.

We applied the validated model to characterize dynamic range effects across the full patient cohort (*N* = 101) and diverse respiratory parameters. Decreasing dynamic range values systematically reduced beam‐on time across all treatment fields, Figure 1, exhibiting a sigmoid‐shape pattern. Plateau regions at the extremes (dynamic range near 10 or 200) indicate that machine dose rate limits were reached across energy layers. However, the intermediate region (dynamic range from 50 to 150) showed a rapid reduction, consistent with the dose rate elevation mechanism described in Section 2.2 and previous findings.^14^

**FIGURE 2:**
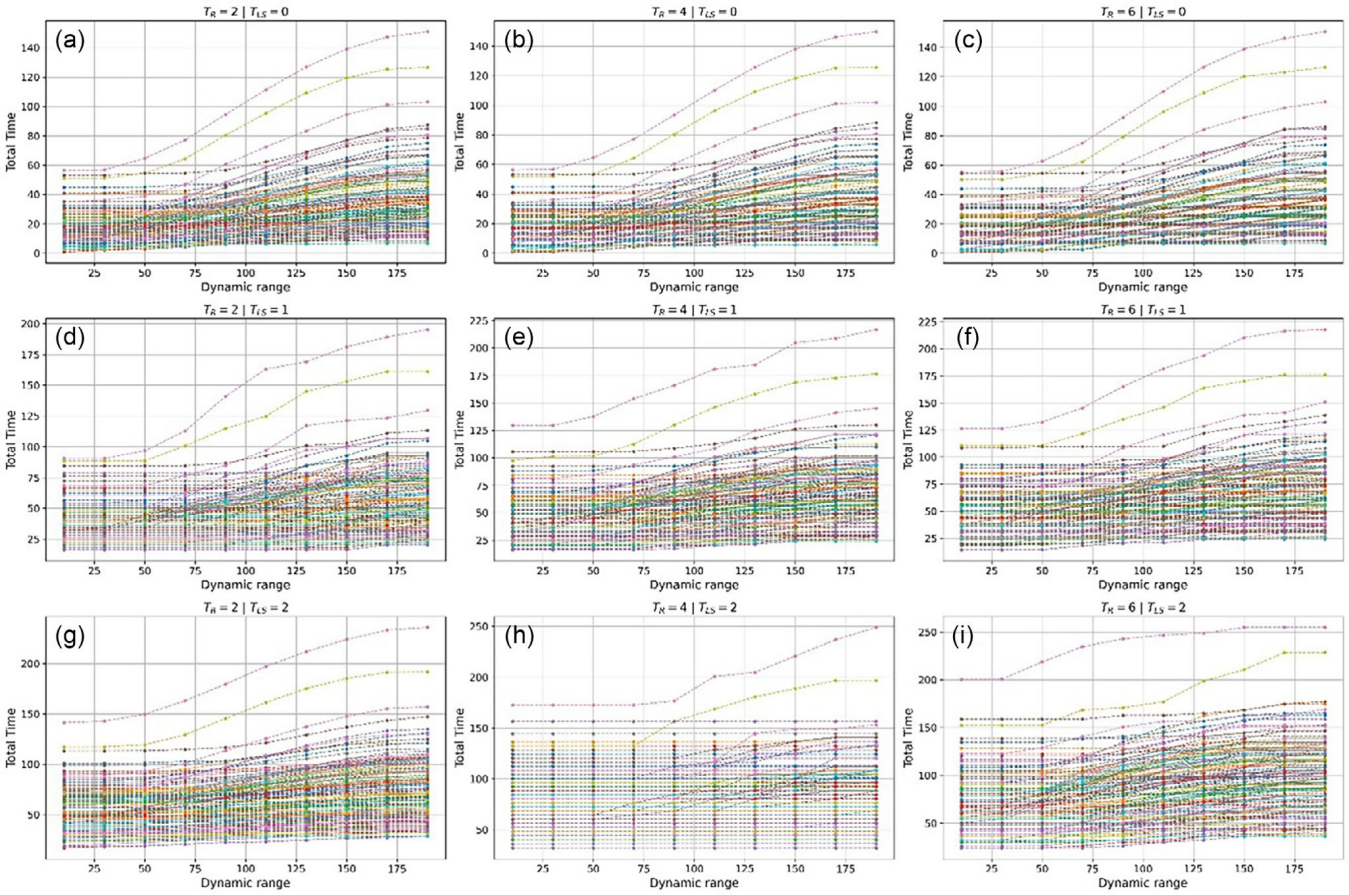
Treatment time versus dynamic range for different TLS values. (a–c) *T_LS_* = 0 s, (d–f) *T_LS_* = 1 s, (g–i) *T_LS_* = 2 s. Plateaus indicate dynamic range thresholds below which no efficiency gain occurs.

Figure 2 reveals complex interactions of dynamic range and respiratory gating. Without layer switching (*T_LS_* = 0 s), total treatment time behaves as a sigmoid function of dynamic range values, similar to Figure 1. Notably, at *T_LS_* = 2 s and *T_R_* = 4 s, most fields have a constant total treatment time across varying dynamic range values. This reflects the synchronization between respiratory gating and layer delivery timing, as found in our previous study.^3^ Dynamic range‐mediated beam‐on time reduction provides no benefit when layer delivery plus switching completes within a single gating‐on period, forcing the system to wait for subsequent respiratory cycles.

**FIGURE 3:**
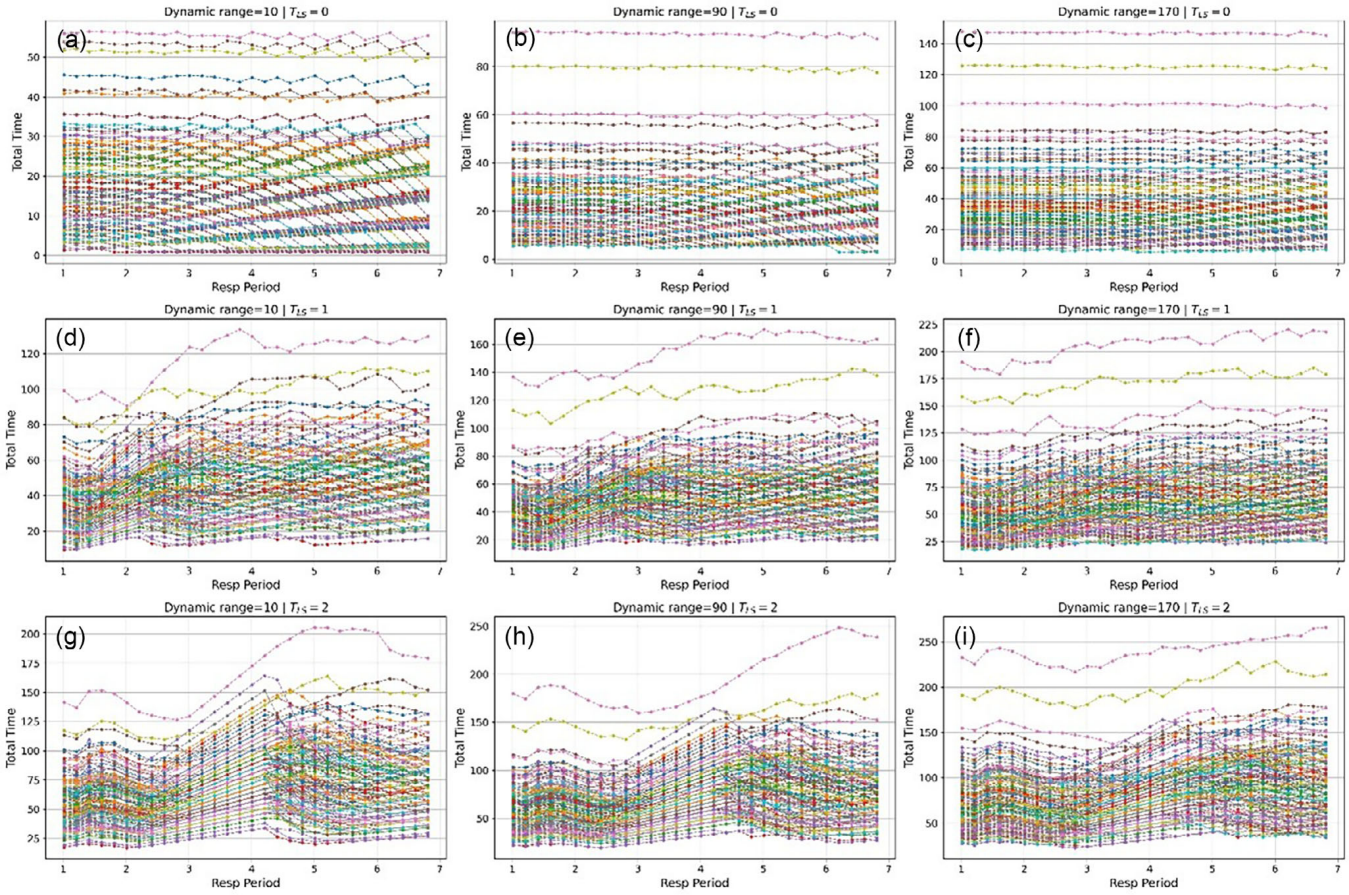
Total treatment time versus respiratory period for different T_LS_ values. (a–c) *T_LS_* = 0 s, (d–f) *T_LS_* = 1 s, (g–i) *T_LS_* = 2 s. Peaks near *T_R_* = 4 s reflect gating‐delivery synchronization effects.

Figure 3 demonstrates that treatment time reaches plateaus at dynamic range thresholds that are dependent on the respiratory period. At *T_LS_* = 2 s, plateaus appear most consistently at *T_R_* = 4 s, confirming that this respiratory period maximizes delivery efficiency under gating constraints, as found in our previous study.^3^ However, at lower dynamic range values, total treatment time tends to be constant across the dynamic range values. This means that the effect of synchronization no longer helps reduce the total treatment time, or it has reached a plateau, which is consistent with Figure 2. The result is also illustrated in a violin plot (Figure S5).

**FIGURE 4:**
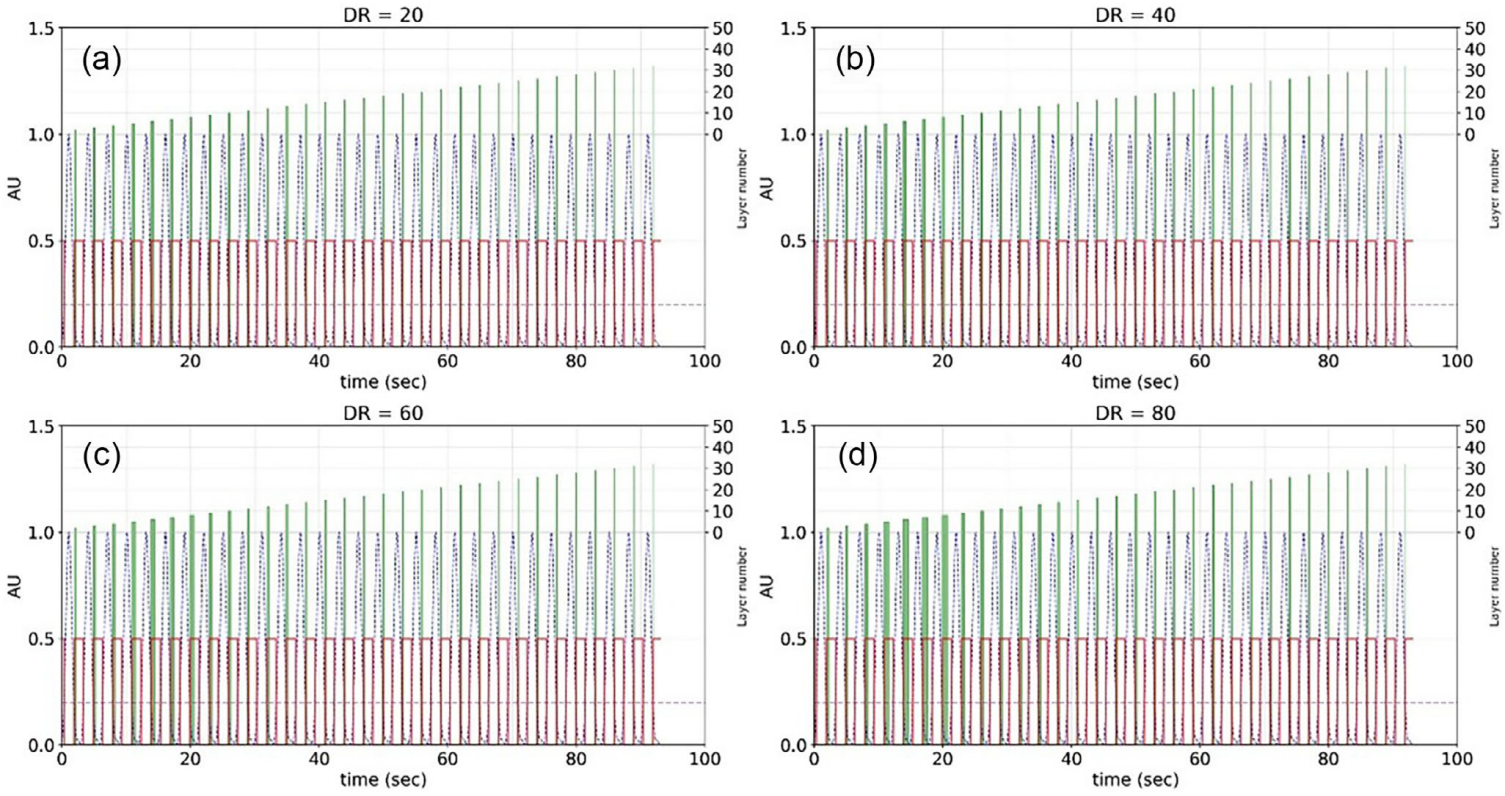
An illustration of the plateau mechanism. The value of dynamic range is (a) 20, (b) 40, (c) 60, and (d) 80. Green rectangles: layer beam‐on time; despite reduction from Panels D–A, total treatment time remains 92 s due to gating‐delivery synchronization.

Figure 4 illustrates the plateau mechanism using a representative case. Despite the progressive beam‐on time reduction from dynamic range 80 to 20 (as shown in Panel D to A), total treatment time remained constant at 92 s because layer delivery plus switching completed within the gating‐on periods. Further reduction in beam‐on time cannot affect the total treatment time. Therefore, plateau identification is needed to prevent unnecessary dosimetric compromise resulting from excessive dynamic range reduction.

### 3.3 Dosimetric quality trade‐offs

We quantified dosimetric costs through two fully optimized plans in RayStation v2025 for Patient 14, differing only in dynamic range constraint: baseline (no dynamic range, RayStation v6), dynamic range = 100, and dynamic range = 10.

**FIGURE 5:**
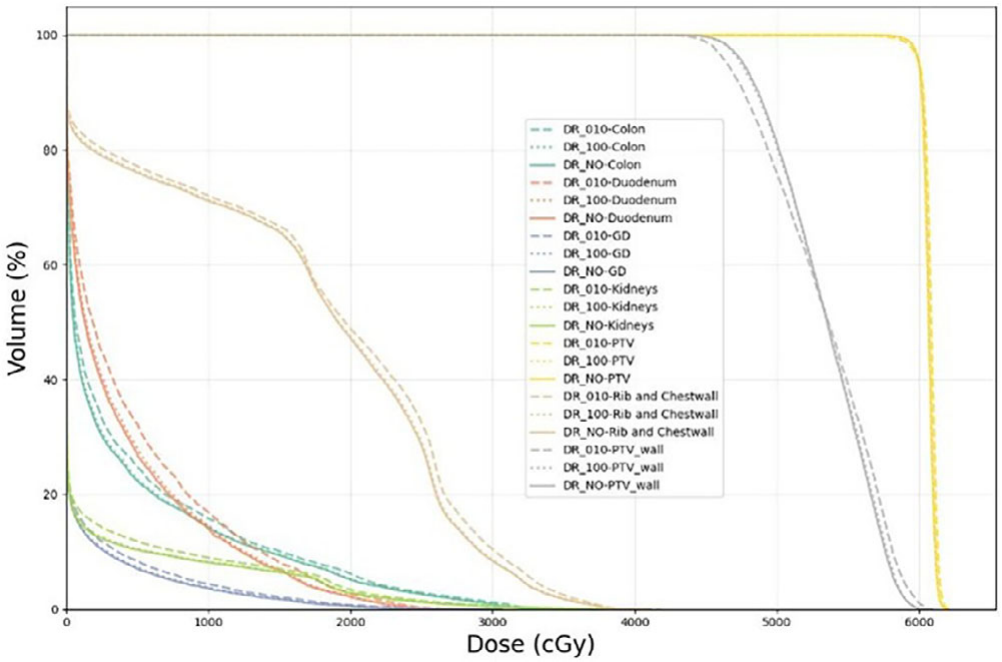
Dose‐volume histograms for three independently optimized plans: baseline (green, no dynamic range), dynamic range = 100 (orange), dynamic range = 10 (blue). All used identical dose constraints.

Figure 5 and Table 3 show that target coverage remained equivalent across dynamic range values (PTV mean dose: 6080.1 vs. 6066.1 cGy for dynamic range = 10 vs. baseline, 0.23% difference). However, normal tissue doses increased systematically with lower dynamic range values. Organs proximal to the target experienced the largest relative increases: gallbladder mean dose increased 16.36% (16.8 cGy absolute), duodenum 16.34% (63.7 cGy), kidneys 12.91% (24.4 cGy), and chest wall 3.55% (59.9 cGy). These dose escalations result from reduced intensity modulation capability. Lower dynamic range values constrain the optimizer's ability to deliver very low MU values typically used for normal tissue sparing. RayStation v2025 is adapted by adjusting spot weights and potentially modifying spot positions within energy layers. PTV wall analysis revealed conformality degradation (mean dose: 5325.8 vs. 5333.5 cGy). While absolute increases remained clinically acceptable for most organs (at prescription 6000 cGy, dose difference is less than 100 cGy for normal organs), the consistent pattern warrants attention when selecting dynamic range values, particularly for patients with organs‐at‐risk adjacent to targets.

**TABLE 3:** Dosimetric comparison of fully optimized plans with different dynamic range constraints. Percentages show changes from baseline. Plans represent actual RayStation v2025 optimization, not model predictions.

ROI	Plan	D_Avg_ (cGY)	D_med_ (cGY)	D_Max_ (cGY)
Colon	DR_010	421.8 (9.42 %)	740.8 (5.36 %)	3671.1 (0.59 %)
	DR_100	391.2 (1.48 %)	703.8 (0.10 %)	3641.0 (–0.24 %)
	DR_NO	385.5	703.1	3649.7
Duodenum	DR_010	453.6 (16.34 %)	730.8 (10.98 %)	3015.4 (1.52 %)
	DR_100	401.2 (2.90 %)	675.9 (2.64 %)	2955.9 (0.48 %)
	DR_NO	389.9	658.5	2970.3
GD	DR_010	119.5 (16.36 %)	326.8 (11.16 %)	3015.4 (3.09 %)
	DR_100	105.7 (2.92 %)	298.7 (1.60 %)	2955.9 (1.05 %)
	DR_NO	102.7	294.0	2925.1
Kidneys	DR_010	213.4 (12.91 %)	486.3 (12.57 %)	4114.9 (3.95 %)
	DR_100	191.9 (1.53 %)	436.4 (1.02 %)	3998.7 (1.01 %)
	DR_NO	189.0	432.0	3958.7
Chest wall	DR_010	1749.4 (3.55 %)	2020.9 (2.40 %)	4143.0 (2.91 %)
	DR_100	1699.2 (0.57 %)	1981.4 (0.40 %)	4038.7 (0.32 %)
	DR_NO	1689.5	1973.5	4026.0
PTV	DR_010	6080.1	5983.0	6228.4
	DR_100	6066.6	5988.4	6213.0
	DR_NO	6066.1	5984.7	6198.4
PTV wall	DR_010	5325.8	4926.2	6102.3
	DR_100	5335.1	4977.8	6055.6
	DR_NO	5333.5	4986.2	6054.0

### 3.4 Python script for clinics

**FIGURE 6:**
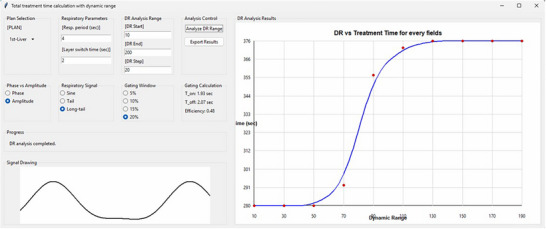
Clinical tool interface showing treatment time versus dynamic range. Red dots: calculations; blue line: interpolation. User inputs patient‐specific parameters (*T_R_* = 4 s, *T_LS_* = 2 s shown).

We developed a RayStation script (Txtime_DR, available at GitHub: https://github.com/jokh38/Txtime_DR) that integrates into RayStation v2025 workflows. Figure 6 demonstrates the tool's interface using a representative case. This tool accepts user‐defined parameters (*T_R_*, *T_LS_*, dynamic range, and gating specifications) and provides treatment time predictions across dynamic range parameter space, identifies efficiency plateaus where further dynamic range reduction provides no benefit, and determines optimal patient‐specific dynamic range values balancing treatment time and dosimetric quality.

## 4 DISCUSSION

Our computational framework enabled the systematic exploration of dynamic range‐respiratory interactions across 101 patients. Validation results established reliable performance, indicating our model captures the essence of the behavior related to dynamic range values in RayStation v2025. Therefore, this study established dynamic range as a planning‐based optimization strategy for respiratory‐gated proton therapy.

Our study assumes a fixed energy layer configuration and line segment distribution. The validation results demonstrate that redistribution of line segments is not frequent, and it is aligned with our assumption. Based on this result, we propose a clinical decision framework: (1) Identify the dynamic range threshold where treatment time plateaus using our RayStation‐integrated Python script; (2) Generate a fully optimized plan with this dynamic range value; (3) Evaluate whether normal tissue dose increases remain clinically acceptable; and (4) Select the optimal dynamic range value that balances efficiency and dosimetric quality.

Notably, full re‐optimization at the selected dynamic range value may redistribute line segments differently than predicted, potentially affecting normal tissue doses. For patients with organs‐at‐risk adjacent to targets, higher dynamic range values may be necessary to maintain acceptable dose distributions, even at the cost of modest efficiency gains. The user should evaluate the fully optimized plan quality in RayStation v2025 when using dynamic range values in the process.

In our previous work, we established that the beam‐on time efficiency is maximized with the relationship between the respiratory period and layer switching time, *T_R_^M^* = a∙*T_LS_* + b.^3^ For example, the condition (*T_R_* = 4 s, and *T_LS_* = 2) represents a maximized efficient case, as illustrated in Figure 2h of this study. However, unlike the findings of the previous study, Figure 2h shows that the total treatment time did not change across the dynamic range values. Although this result appears contradictory, it remains aligned with our previous conclusion. The fundamental reason for the maximization under this relationship is the optimal synchronization between the gating‐off period and layer switching. When this synchronization occurs, further reduction in beam‐on time alone cannot decrease the total treatment time, as shown in Figure 4. This observation reinforces our previous conclusion.

Dynamic range optimization can be naturally combined with other efficiency strategies, including spot optimization, spot number reduction, delivery pattern optimization, and beam sequencing optimization. The synergistic combination may achieve greater treatment time reductions, though cumulative dosimetric impact requires systematic evaluation. Furthermore, treatment time reductions enable higher patient throughput, potentially improving proton therapy access. Shorter treatment time also reduces patient discomfort and intrafractional motion, particularly beneficial for elderly patients or those with compromised respiratory function.

Our treatment time predictions employed synthesized sinusoidal breathing signals to demonstrate the effect of dynamic range optimization on total treatment time. We emphasize that dynamic range optimization is used in concert with respiratory gating, not as a replacement. Moreover, this method does not require synchronization between breathing and energy layer switching. Dynamic range constraints reduce beam‐on time by increasing minimum MU values per energy layer, an operation that is independent of the breathing phase. Even with irregular breathing patterns, the dynamic range‐optimized plan delivers the prescribed dose with reduced beam‐on time during each gated interval, resulting in shorter total treatment time.

The magnitude of time reduction may vary with gating efficiency. Patients with highly irregular breathing may experience lower gating efficiency, leading to limited absolute time savings. However, dynamic range optimization remains beneficial: for any given gating efficiency, a dynamic range‐optimized plan requires less beam‐on time than a non‐optimized plan. Our proposed clinical decision framework recommends evaluating this trade‐off on a case‐by‐case basis.

Our method is valid under the assumption of “fixed energy layer configuration and line segment distribution” across different dynamic ranges. While the detailed numbers may vary, these variations act as a normalization factor that does not affect the overall beam‐on time versus dynamic range relationship. The validation in Section 3.1 demonstrates that our method is valid when this assumption holds. However, this validation was performed for a single patient case, which confirms the validity of our method under the stated assumption rather than across the entire parameter space.

Our method may deviate from fully optimized plans in RayStation v2025 when the assumption does not hold, which could occur at extremely low dynamic range values. However, such extreme settings may not be clinically beneficial due to the plateau behavior observed in beam‐on time reduction. Therefore, we recommend using our Python tool as guidance for treatment planning optimization. The final treatment plan should be evaluated in RayStation v2025 for both efficiency gains and dosimetric quality before clinical implementation.

Future research should integrate dynamic range optimization into automated treatment planning workflows^15^ and develop machine learning algorithms that balance efficiency and quality based on patient‐specific anatomical features.^16^ The relationship between dynamic range optimization and emerging technologies such as real‐time adaptive planning deserves investigation.^17^ Clinical implementation requires prospective validation through systematic monitoring of treatment outcomes and patient tolerance. Our RayStation‐integrated tool provides the technical foundation for routine clinical adoption, enabling individualized dynamic range selection during treatment planning without additional computational burden.

## 5 CONCLUSION

This study established dynamic range as a planning‐based optimization strategy for respiratory‐gated proton therapy that reduces treatment time without requiring further patient cooperation or system modifications. Our computational framework, validated against RayStation v2025 (error < 10.4%), accurately predicts treatment time across diverse respiratory patterns and identifies patient‐specific dynamic range thresholds where efficiency plateaus occur. Dosimetric evaluation using fully optimized plans quantified the trade‐off: lower dynamic range values reduce treatment time but systematically increase normal tissue doses while maintaining target coverage.

Dynamic range optimization operates within respiratory gating constraints rather than replacing gating. Respiratory gating remains active throughout treatment delivery to manage intrafractional motion, while dynamic range pre‐optimizes beam delivery efficiency during gating‐on periods as a planning parameter. This separation enables treatment time reduction across patient populations regardless of breathing regularity.

A RayStation‐integrated tool enables clinical implementation by evaluating treatment time reduction and dosimetric impact for individual patients, facilitating dynamic range selection that balances efficiency gains against acceptable normal tissue exposure. This approach addresses treatment time challenges in respiratory‐gated proton therapy through immediately accessible planning‐system functionality.

## AUTHOR CONTRIBUTIONS

**Sungkoo Cho**: Data curation, Writing—Original Draft. **Jung Il Yu**: Investigation. **Hee Chul Park**: Data Curation. **Kwanghyun Jo**: Conceptualization, Supervision, Project Administration, Validation, Writing—Review and Editing.

## CONFLICT OF INTEREST STATEMENT

The authors have no relevant financial or non‐financial interests to disclose.

## Supporting information



Supporting information
