# Supplementary material for: Dynamic range optimization for treatment time reduction in respiratory‐gated proton therapy using RayStation v2025
Source: J Appl Clin Med Phys. 2026 Feb 16;27(2):e70510. doi: 10.1002/acm2.70510 (PMC12909597; doi:10.1002/acm2.70510)
Supplement: Supplementary file 1 — Supporting information [file ACM2-27-e70510-s001.docx]

**S1 | Materials and methods**

**S1.1 | Patient characteristics and treatment planning**

Table S1 Characteristics of treatment plan.

| **Characteristics** | **N (%)** |
| --- | --- |
| **Tumor volume (cc)**  0 - 100  100 – 200  200 – 300  300 – 400  400 – 500  500 – | 54 (53.4)  20 (19.8)  12 (11.9)  7 (6.9)  3 (3.0)  5 (5.0) |
| **Fraction size (cGy)**  300 – 400  400 – 500  500 – 600  660 | 8 (7.9)  4 (4.0)  16 (15.8)  73 (72.3) |
| **Number of fields**  2  3 | 51 (50.5)  50 (49.5) |
| **Number of layers**  0 – 10  10 – 20  20 – 30  30 – 40  40 – 50 | 3 (1.2)  96 (38.1)  120 (47.6)  29 (11.5)  4 (1.6) |
| **Scan time (sec)**  0 – 10  10 – 20  20 – 30  30 – 40  40 – 60  60 – | 67 (26.6)  108 (42.9)  48 (19.0)  18 (7.1)  7 (2.8)  4 (1.6) |

**S1.2 | The BoT calculation with implementation of DR**

The BoT calculation for continuous line-scanning involves multiple processes that must be constrained within the machine limits of scanning speed and dose rate. The scanning speed of the proton beam is determined by the magnetic field strength of the scanning magnet in the nozzle. The scanning speed ranges from 0.1 cm/s to 2000 cm/s. The dose rate is limited by cyclotron and energy selection system, with a lower limit of 1.4 MU/s and an upper limit that depends on proton energy. The energy selection system uses a thick degrader, resulting in a lower dose rate for low-energy proton beams compared to high-energy proton beams. Specifically, the dose rate is 3.91 MU/s for 70 MeV and 20 MU/s for proton energies above 146 MeV.

**S1.3 | Total time and BoT efficiency calculation**

BoT efficiency, denoted as Eff, is defined as the ratio of the delivery time without gating (T_BoT_ + T_LS_ x (N_Layer_ -1)) to the total delivery time with gating (T_Total_):

$$Eff=\frac{T_{BoT}+T_{LS}\times\left( N_{Layer}-1 \right)}{T_{BoT}+T_{LS}\times\left( N_{Layer}-1 \right)+ T_{dead}}, (S1)$$

This definition of BoT efficiency aligns with the general concept of efficiency as the ratio of treatment times with and without respiratory influence. In proton therapy, the treatment time without respiratory effects must include T_LS_s, as reflected in the numerator of Equation (3). Therefore, we define BoT efficiency for proton therapy using Equation (S1).

**S2 | Results**

**S2.1 | BoT efficiency calculation**

Figure S**1** displays the calculated BoT efficiencies for all fields as a function of DR. We set T_LS_ to 1 second for panels A through F, and to 2 seconds for panels G through L. Note that TLS = 0 is not presented because it has a trivial behavior – it has the same value for the whole range of DR. Based on the total treatment time results from the previous section, we expected that smaller DR values would increase BoT efficiency. This expectation holds true for panels A, G, and H; however, panels B and J demonstrate the opposite trend. This counter-intuitive behavior occurs when 0.5 × T_R_ ≈ T_LS_, meaning the gating-on time approximately equals both the gating-off time and T_LS_. These results are closely related to our previous conclusion that maximum efficiency is achieved when the respiration period follows T_R_^M^ = a × T_LS_ + b, such as (TR = 2, TLS = 1) and (TR = 4, TLS = 2). The efficiency behavior contradicts our initial expectations, and it will be discussed in detail in the following sections.

**
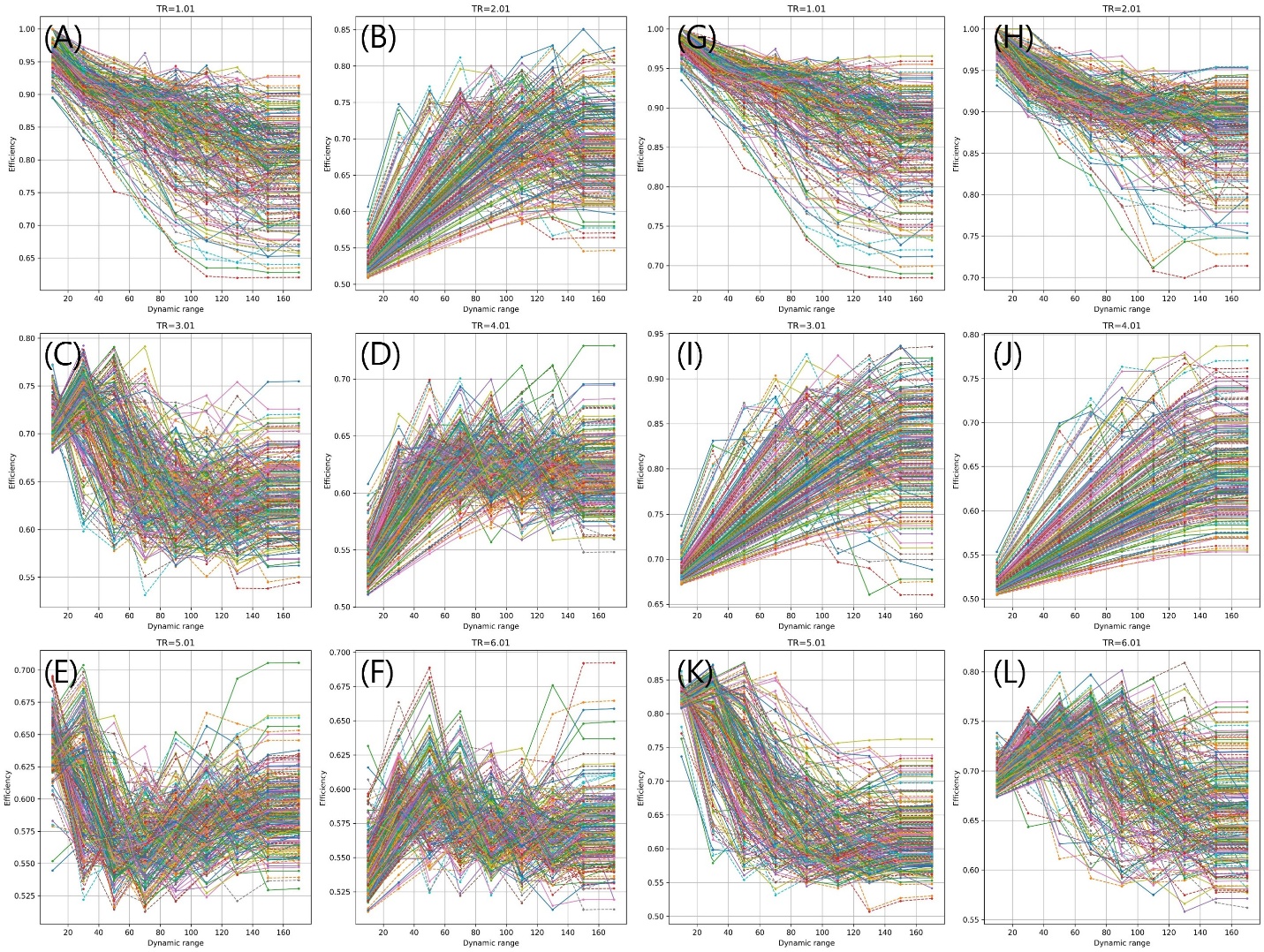
**

Figure S1 Efficiency vs DR for TLS = 1 (A to F) and 2 (G to L) seconds

To compare with our previous work, Figure S**2** is presented, showing BoT efficiency as a function of T_R_. Since the maximum DR value is 200, the graph at DR = 170 closely resembles the results of our previous work. However, at smaller DR values, every field exhibits similar behavior, as demonstrated in the DR = 10 case. This convergence occurs because shorter BoT durations are completed within individual respiration cycle, making the gating-off period as the primary contributor to total treatment time across all fields.


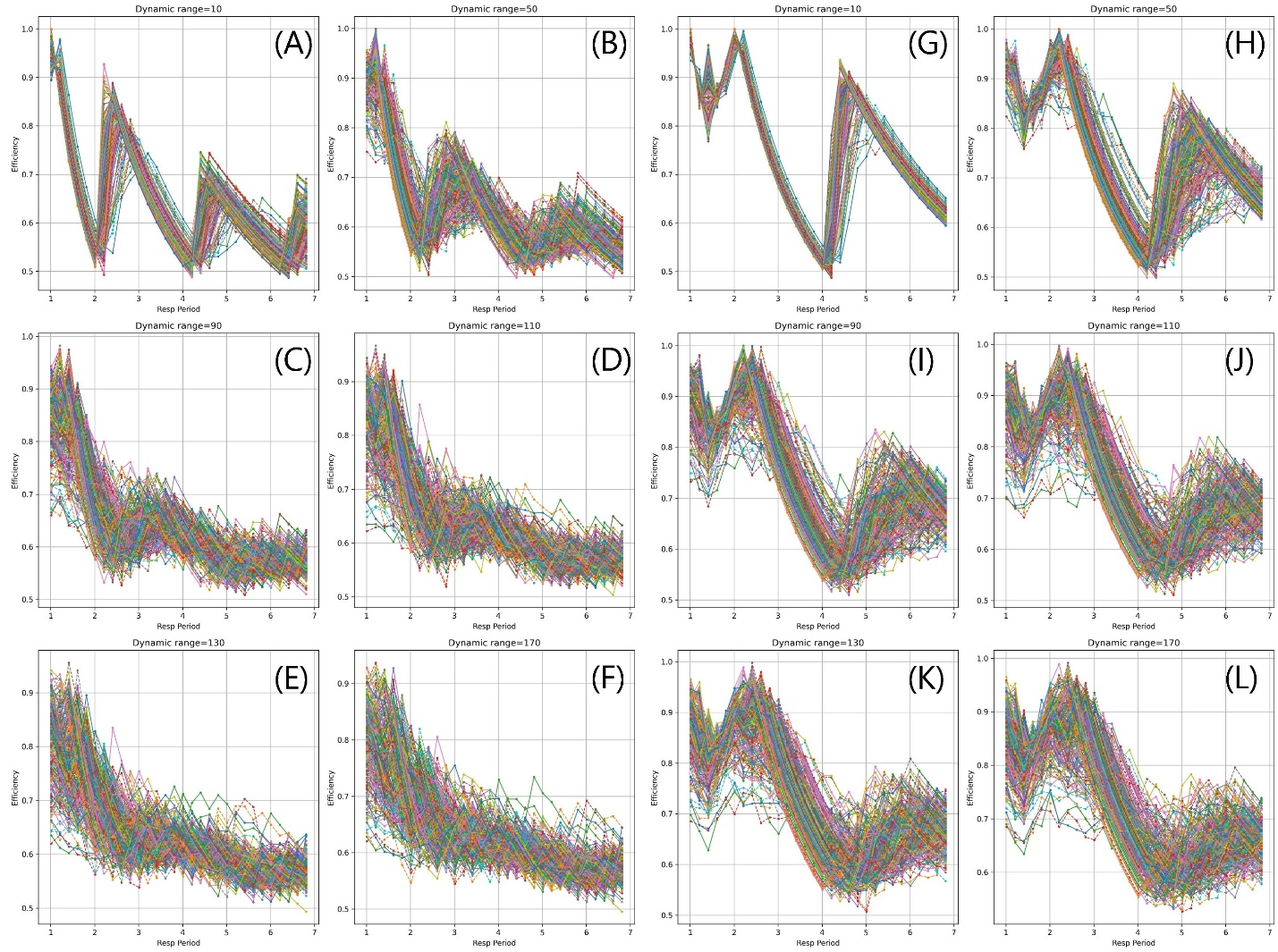


Figure S2 Efficiency vs TR for TLS = 1 (A to F) and 2 (G to L) seconds

**S2.2 | Beam-on Time Reduction Analysis**

Figure S3 provides a layer-by-layer visualization of the dynamic range (DR) constraint effects on the beam-on time (T_BoT_) for the representative case.

- Beam-on Time Difference (Panels A and C): These panels cumulatively show the reduction in beam-on time (BoT) relative to the baseline (DR NO) plan for DR values of 200, 100, 50, and 10 across each energy layer. A larger negative difference indicates greater BoT reduction. The most pronounced reduction occurs in the middle energy layers (approximately layers 10-25), suggesting these layers originally had the widest monitor unit (MU) range and were thus most significantly impacted by the DR-imposed elevation of the minimum MU threshold.
- Beam-on Time Ratio (Panels B and D): These log-scale plots illustrate the proportional reduction in BoT for each layer. The consistent pattern across layers, where a lower DR value leads to a dramatically lower time ratio, confirms that the DR mechanism effectively increases the layer-specific dose rate, thereby shortening the beam delivery duration for a fixed dose.

**S2.3 | Stability of Line Segment Configuration**

Figure S4 presents an analysis of the change in the number of line segments for plans fully re-optimized in RayStation v2025 under different DR constraints, compared to the baseline plan.

- Difference in Line Segment Count: The bars represent the difference in the number of line segments per energy layer for DR values of 10, 50, 100, and 200 relative to the baseline (DR NO) plan.
- Validation of Model Assumption: The analysis confirms that minimal change in the number of line segments occurred across the majority of energy layers and ports. Specifically, a difference of 2 segments was observed in layer 25 for Port 1, and a maximum difference of 2-3 segments was found in the final layer (layer 37) for Port 2. This systematic consistency supports the core assumption of the computational model, which posits that the primary effect of DR is to elevate minimum MU values to increase the dose rate, rather than fundamentally restructuring the spot map or energy layer configuration. This result validates that the model accurately captures the mechanistic essence of DR effects on beam-on time.

**
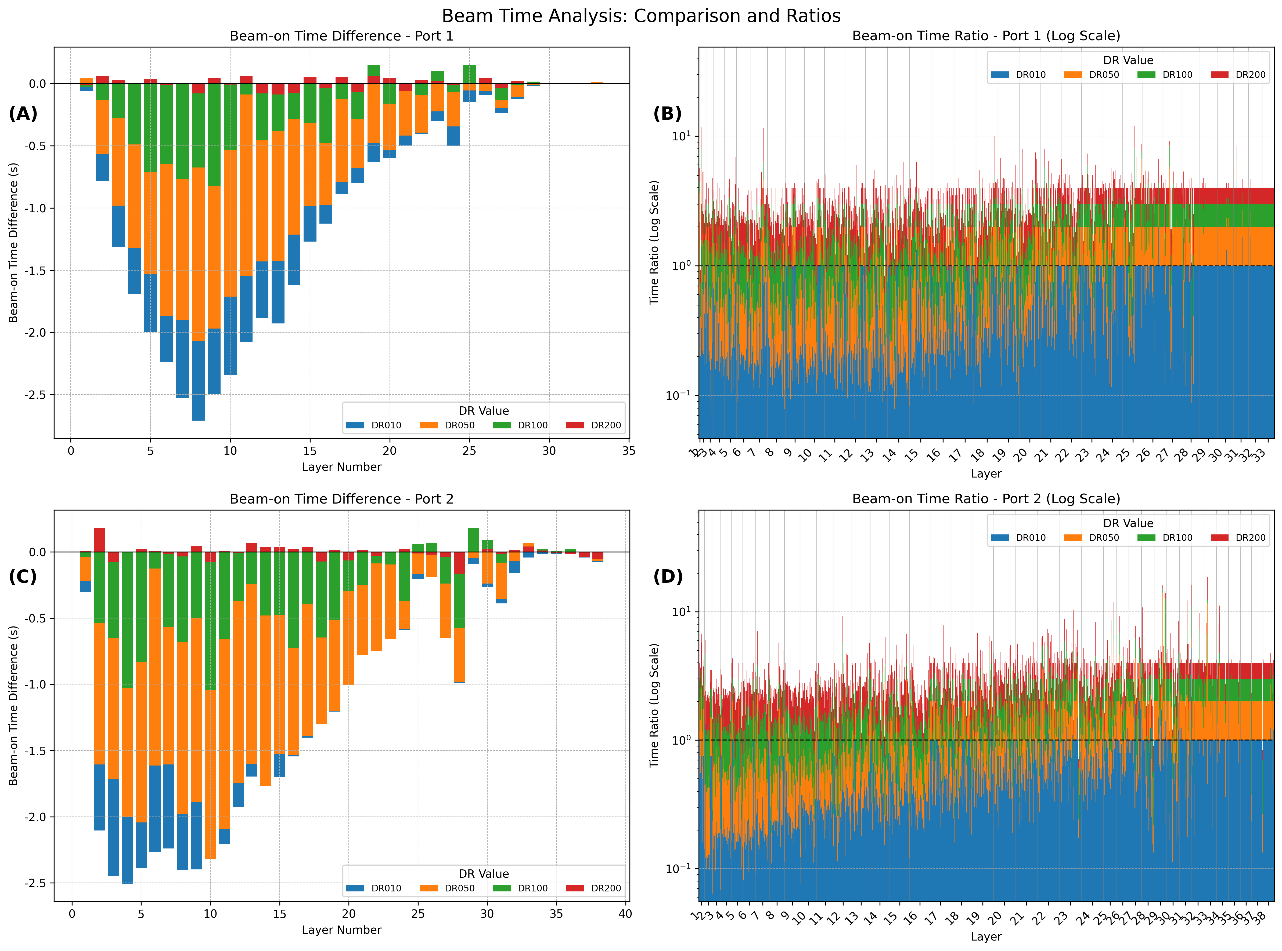
**

Figure S3 Beam-on Time Analysis: Comparison and Ratios. A: Beam-on time difference (seconds) for Port 1(2) across energy layers. B: Beam-on time ratio (log scale) for Port 1 (2) across energy layers. The stack of bars in (A) and (C) and the ratios in (B) and (D) illustrate the layer-specific beam-on time reduction achieved by progressively lowering the Dynamic Range (DR) value from 200 to 10. Every plan were optimized in RayStaion v2025.

**
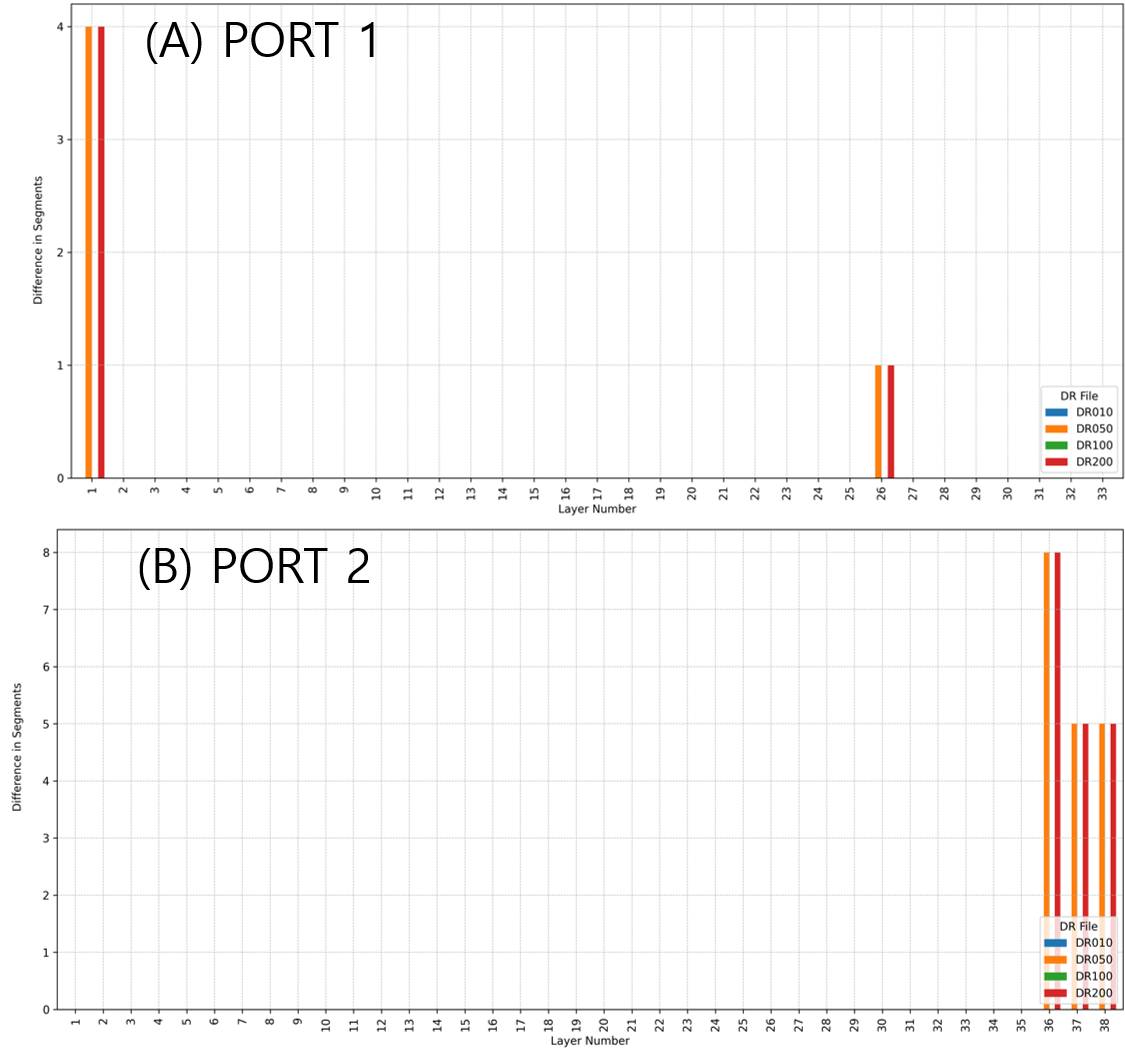
**

Figure S4 The difference in the number of line segments for every layer. Difference in the number of line segments per layer for Port 1 (A) and 2 (B). The data compares the segment counts in fully re-optimized plans (DR=10, 50, 100, 200) against the baseline plan (DR NO). Minimal differences in segment counts confirm the model's assumption that dynamic range primarily modifies minimum Monitor Unit (MU) values, thereby increasing the layer dose rate, rather than altering the beam delivery pattern's structure.

**S2.4 | Violin plot of efficiency gain**

Figure S5 represents the analysis of efficiency ratios achieved through dynamic range optimization. The efficiency ratio is defined as the relative ratio between the total treatment time (at maximum optimized with dynamic range 10) divided by the baseline time (dynamic range 190). A ratio lower than 1.0 indicates a reduction in total treatment time due to dynamic range effects. The violin plots display the distribution of these ratios, with horizontal lines indicating the quartiles and the width representing the frequency density.

For T_LS_ = 0.5 seconds, a consistent time reduction of approximately 30% (ratio ~ 0.7) is observed across all respiratory periods (T_R_). However, minimal efficiency gains (ratio approaching 1.0) are observed at specific (T_LS_, T_R_) pairs: (1, 2), (1.5, 3), and (2, 4). These points correspond to the optimal synchronization conditions identified in Figure S2 and our previous work [3]. At these synchronization points, the baseline delivery is already highly efficient, leaving little margin for further time reduction via dynamic range optimization.


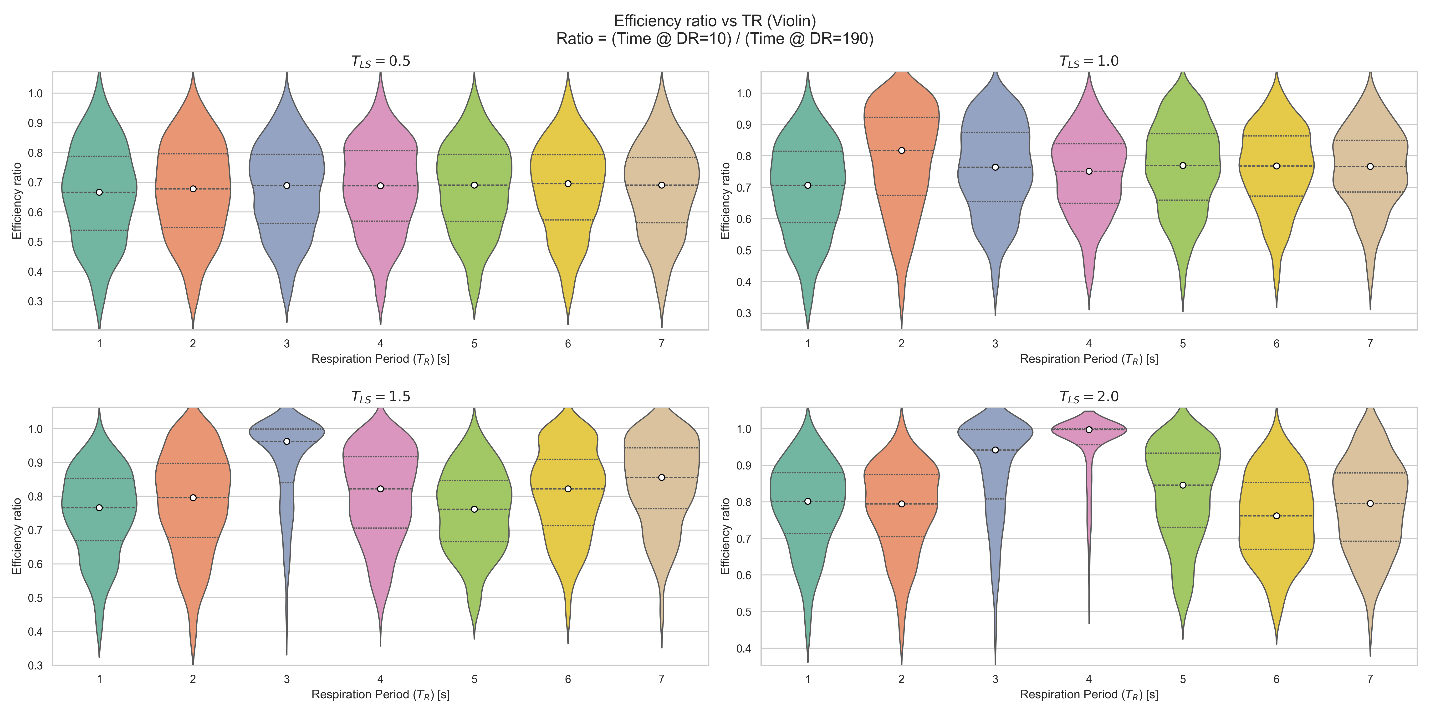


Figure S5. Violin plots of the relative treatment time ratio versus respiratory period (T_R_) for various layer switching times (T_LS_). The ratio is defined as Time (DR = 10) / Time (DR = 190). Horizontal lines within each violin represent the lower, median, and upper quartiles. A lower ratio indicates a greater reduction in treatment time. Note that at synchronized points (e.g., T_LS_ = 1 second, T_R_ = 2 seconds), the ratio approaches 1.0, indicating minimal benefit from dynamic range optimization due to the already maximized baseline efficiency.
